# Some Observations on the Clinical and Pathological Distinction between Phthisis and Tuberculosis

**Published:** 1878-01

**Authors:** Wm. T. Belfield

**Affiliations:** House Surgeon, Cook County Hospital


### Chicago Medical Journal

AND

### EXAMINER.

VOL. XXXVI.—JANUARY, 1878.—NO. 1.

### Original Communications.

### THE PATHOLOGICAL TRANSACTIONS OF THE CHICAGO MEDICAL SOCIETY.

EDITED BY DR. I. N. DANFORTH.

I.

### SOME OBSERVATIONS ON THE CLINICAL AND PATHOLOGICAL DISTINCTIONS BETWEEN PHTHISIS AND TUBERCULOSIS. BY DR. WM. T. BELFIELD, House Surgeon, Cook County Hospital.

That phthisis and tuberculosis are clinically and pathologically distinct, receives general assent, if I mistake not, in Germany. In France, the recent investigations of Charcot, though not yet confirmed, have, by the sheer weight of that illustrious name, shaken the Gallic faith in the same creed. In the United States the great majority of native-born professional men reject the duality of the diseases. It is my purpose in this paper, not to discuss one or the other theory, but simply to present some observations made in the wards of the County Hospital, which seem to me to argue duality in both clinical history and pathology.

I present, first, cases of acute tuberculosis, which embody four facts of interest:

1*st.* That the constitutional symptoms, which indicate grave disturbance, are disproportionate to the physical signs, which, alone, would suggest a trifling lesion.

2*nd.* That the accompanying fever is of the continued type, the thermometer showing but little variation, morning or evening.

3*rd.* That the tuberculosis follows, at a greater or less interval, a local inflammation, whose products have undergone degenerative changes.

4*th.* That the characteristic lesion is the production of a neoplasm without, necessarily, subsequent disintegration of the lung tissue.

CASE I.—Frank B., aged 24, a German carpenter, was admitted to the hospital March 12, 1877. He was greatly emaciated and very feeble; exhibited a marked febrile action — pulse 120, temperature 104, respiration 52. There was marked dyspnœa and cyanosis, the blueness being very evident even under the nails; he had a constant cough and profuse whitish expectoration. So precious was his scanty breath, that he could scarcely tell us the following history.

There was no record of consumption in his family. His own health had been excellent until March, 1876, when he began to suffer from loss of appetite and great pain in the abdomen. In a few weeks there appeared several tumors, which could be plainly felt in the umbilical region. During the summer of 1876 he regained fair health, though the tumors never decreased in size. His health remained good until about January 1, 1877, when it declined; had pain in his chest; cough; expectoration of white, frothy sputum; shortness of breath; fever; began to lose flesh and strength. His illness, while steadily increasing, had been especially aggravated during the three weeks prior to admission.

On physical examination, we found the form and movements of the chest and the percussion resonance normal; exaggerated breathing, dry and moist râles over the entire chest. There was nowhere any evidence of pulmonary consolidation.

During his stay in the hospital, the prominent features of his case remained unchanged. His pulse, temperature and respirations showed but slight variation—morning or evening. The extreme dyspnœa and cyanosis persisted until death, which occurred two days after admission.

An examination was made ten hours after death. The two layers of pleura were adherent throughout. Each lung was thickly studded with miliary tubercles, evenly disseminated from apex to base. There was nowhere any sign of recent or ancient consolidation or destruction of lung-tissue. Many of the mesenteric glands were much enlarged and caseous. One aggregation of them constituted a tumor as large as an orange, and there were several smaller tumors of similar composition. The other organs appeared normal.

Case II.—J. K., an English carpenter, aged 52, was admitted August 20, 1877, and gave the following history: Had no hereditary taint; had always enjoyed excellent health. On the 20th of April last he had suffered a thorough drenching. Next day he had a chilly feeling, fever, slight cough and sharp pain in left side. In a couple of weeks, however, his symptoms subsided, though he still remained weak. This was his condition until four weeks before admission, when he became feverish and short of breath; his general health declined, and he took to his bed, thinking that he was suffering from asthma.

On admission, he was quite emaciated, and presented the usual symptoms of a febrile condition; pulse 136; temperature 102 (evening); respirations 48. Found evidences of large effusion into left pleural sac, and compression of left lung; exaggerated breathing on the right side. On the next day (August 21) 60 ounces of straw-colored liquid were withdrawn by means of the aspirator. Patient at once expressed great relief; his respirations fell from 48 to 16 per minute; his temperature next morning was only 99½°. On August 23, however, his pulse was 126, temperature 103, respirations 26, and he was suffering considerable dyspnœa. Notwithstanding the free use of stimulants, he steadily failed, and died August 29. During the four days preceding his death, his pulse ranged from 112 to 140, his respirations from 26 to 34; his temperature averaged in the morning 103½°, in the evening 101°.

An examination was made 12 hours after death. The left lung was found crowded into the upper fourth of the left thorax; the lower three-fourths of the cavity was converted into a shut sac by means of a pseudo-membranous exudate which lined the thoracic walls and was thence reflected on to the lower surface of the compressed lung above and on to the upper surface of the diaphragm below. Within this sac were about 40 ounces of pale liquid. Both lungs were thickly and uniformly studded with miliary tubercles. Other organs about normal.

CASE III.—Was mentioned by Dr. Meyer in the CHICAGO MEDICAL JOURNAL AND EXAMINER for April, 1877.

E. L., a Norwegian silversmith, about 26 years old, was admitted December 16, 1876. A few days before his death, the patient, while delirious, destroyed the record of his case. Hence only the general facts of his history can be given.

He had pleurisy some months previously, and, on admission, his right chest presented the physical signs of hydrothorax and of firm pleuritic adhesions. Yet his symptoms indicated more lesions than were accounted for by the physical signs; he had rapid breathing, dyspnœa, cyanosis and pain in the chest. Early in January he became delirious, his delirium presenting a quiet and even melancholy, rather than a violent aspect. During five weeks his symptoms were gradually aggravated, but his physical signs showed little change. Death occurred February 17, 1877.

An examination, made six hours post mortem, showed about three quarts of light yellow liquid in the right pleural sac; the pleura was much thickened and presented several strong bands of adhesion. The right lung was compressed to about one-third its normal size, and thickly studded, from apex to base, with miliary tubercles; there was a small cavity in its apex.

On the left side, the two layers of pleura were adherent throughout; the left lung also was completely filled with miliary tubercles; there were no signs of consolidation in either lung. The membranes of the brain were greatly congested; there was a slight serous effusion into the ventricles. There was also found a deposit of semi-transparent, grey tubercles along the course of the vessels, especially on the convex surface of the hemispheres; a similar deposit, slight in amount, at the base; vascular turgescence of the cerebral tissues.

CASE IV.—Mary N., an Irish domestic, aged 50, was admitted May 23, 1877. Her health had been generally good, though for 20 years she had been much addicted to drink. Three months previous to admission, her abdomen had begun to swell, and had steadily increased in size; it had been the seat of considerable pain and tenderness. Her health had gradually failed. During her two months' sojourn in the hospital, she was tapped three times; the liver dullness was found to be less extensive than in health.

An examination made eight hours after death showed between the two layers on the right side firm adhesions which had undergone calcareous degeneration over a considerable space. The peritoneum was thickly and uniformly studded throughout with miliary tubercles; there were many adhesions among the folds of intestine. The liver was very small and very tough.

I present next three cases of acute phthisis, exemplifying four points of interest and of distinction from those just related:

1*st.* That the constitutional symptoms and physical signs accord in indicating the gravity of the affection.

2*nd.* That the accompanying fever is of a *remittent* type, with marked rise of temperature in the evening.

3*rd.* That the disease cannot be constantly nor uniformly associated with a pre-existing lesion.

4*th.* That the characteristic lesion is disintegration of lung-tissue.

CASE V.—J. B., a colored cook, 36 years of age, entered the hospital September 18, 1877. His health had been uniformly excellent, except that since February last he had had almost continuously a slight cough and expectoration. In April he was treated in the hospital for intermittent fever. At this time his lung sounds were found to be normal.

Ten days before admission his usually slight cough became suddenly much aggravated; he became very short of breath; had severe pain in the left side.

On admission he was well nourished; exhibited a high febrile action; pulse 116; temperature $103\frac{3}{4}$; respirations 32. The physical signs were dullness, bronchial breathing, and bronchophony—plainly indicating complete solidification of the upper lobe of the left lung. For several days the disease presented the usual symptoms of pneumonia, except that at no time was the sputum observed to be rusty. Yet resolution did not occur at the usual time; the temperature remained high, and on September 28 the discovery of elastic fibres in the sputum and of cracked-pot resonance and cavernous breathing, announced the disintegration of the lung.

Since that time he has grown gradually but certainly weaker; the physical signs indicate a progressive destruction of lung-tissue. His temperature has shown the characteristic diurnal variation of acute phthisis — averaging in the morning 99.9°, in the evening 103.1° — an average rise of 3.2° Fahrenheit.

CASE VI.—J. G., an Irish laborer, aged 26 years, was admitted August 15, 1877. There was no record of consumption in his family. His own health had been excellent until January, 1877, when he took a severe cold, and subsequently suffered, for several months, from constant cough with yellowish or whitish expectoration. Had been working steadily during the spring and summer.

A few days before admission he had pain in his left side, and became quite feverish. We found him poorly-nourished and exhibiting a febrile action — pulse 120; temperature 101¾; respirations 48. Over the upper lobe of the left lung were found some loss of motion; slight dullness; broncho-vesicular breathing and crepitant râles.

During the next ten days he presented most of the signs of croupous pneumonia; his pulse ranged from 96 to 144; his temperature from 101¼ to 103½; his respirations averaged about 52. (On the 18th, the breathing had become intensely bronchial; on the 19th, moist râles were audible; on the 20th, the sputum was first observed to be of rusty color.) During the next two weeks the patient presented not the usual signs of resolving pneumonia, but continued dullness and bronchial breathing; moist râles, increasing in size and number; a temperature averaging over 101°; respirations about 45. On September 18, cavernous breathing and cracked-pot resonance were heard below the left clavicle; and on the same day the destruction of lung-tissue was confirmed by the discovery of elastic fibres in the sputum.

From that time his general condition grew gradually worse; the physical signs indicated a progressive destruction of the lung, soon including the other lung also.

His morning temperature averaged 99.6°, his evening temperature 101.3°, an average evening increase of 1.7°.

On October 6th patient left the hospital by his own desire.

CASE VII.—Geo. W., an American merchant, aged 27, was admitted Oct. 17, 1877. There is no record of consumption or other hereditary disease in his family history. His own health has been excellent; he never had any lung trouble, nor even an obstinate cough, until July, 1877; at that time he took cold, and for three weeks had a violent cough and profuse whitish expectoration. These subsided in August. On September 1, he took cold again, while on a lake steamer; had a violent cough; his sputum was profuse and yellow, and often contained a little blood; had frequent chills; cold sweats every night; lost flesh very rapidly, his weight having been reduced from 172 to 135 pounds in five weeks.

On admission, patient was much emaciated; temperature, 103¼; found complete solidification of upper lobe of right lung, except just below second rib, where there was a large cavity. His subsequent history has been that of advanced consumption. A noticeable feature is the marked evening rise of temperature. The average morning temperature from the 17th to the 31st of October, inclusive, was 99.3°; the evening temperature during the same time averaged 103.8°, an average rise of 4.5° F.

These cases are examples of the fact that acute pulmonary tuberculosis is distinct in clinical history and pathology from acute pulmonary phthisis.

*Clinically*, we observe, first, that in acute tuberculosis there is disproportion, in acute phthisis harmony, between the indications furnished by the constitutional symptoms and those suggested by the physical signs.

Second, that in tuberculosis the fever is of the continued type, the temperature being, if not uniform, higher in the morning, and *always* above the normal.

*Pathologically*, we note in tuberculosis the pre-existence of an inflammatory product in a state of degeneration; while in phthisis the inflammation originates *de novo*, by some impression made upon the system from without.

Second, that in tuberculosis the inflammatory process does not, while in phthisis it does, necessarily result in destruction of the pulmonary structure.

In the cases of tuberculosis presented, the degenerated inflammatory products were located either in the mesentery or in the pleural sac. Yet metamorphosed products of inflammation are found in neither of these localities nearly so frequently as in the air-cells of the lungs. It is estimated that one death in six, the world over, is caused by consumption, in the broadest sense of that term. It is further computed that in four-fifths of these cases the initial lesion was a catarrh, either of the bronchi or of the lungs — that is, was an idiopathic inflammation ; and it is the caseous degeneration and disintegration of the inflammatory products of this catarrh that constitute the consumption. Hence, one individual in eight, on the average, has caseous masses in his lungs, sooner or later in his lifetime. Therefore we would expect, *a priori*, that a large majority of the observed cases of tuberculosis proper would occur in phthisical patients. The converse of this proposition would also seem reasonable — namely, that the caseous masses in phthisical lungs frequently give rise to the infection which is manifested in tuberculosis. Now the truth of both these presumptions is familiar to all who have witnessed many *post mortem* examinations. It is the exception, rather than the rule, to find no miliary tubercles in the lungs of persons dead from consumption. True, some of these miliary tubercles may be found undergoing cheesy metamorphosis, and it may be impossible to say just how much of the cheesy matter found in the lung is degenerated epithelium, and how much is degenerated tubercle ; indeed the whole confusion about pulmonary tubercle seems to have resulted from the supposition that *all* cheesy matter found in the lungs was transformed miliary tubercle, by which supposition the products of pulmonary inflammation were completely ignored. Certain it is, however, that pulmonary inflammation does result in cheesy products ; also that in certain cases of consumption, cavities and cheesy masses are found, but no evidence of miliary tubercles.

Now, Niemeyer states that in consumption proper—that is, chronic pneumonia with degeneration of inflammatory products and disintegration of lung-tissue—the thermometer shows an average daily variation of from 1½° to 2°—very seldom less, frequently much more; but that when in consequence of the absorption of inflammatory products, tuberculosis has supervened upon the phthisis, the fever changes from an intermittent or remittent to a continued type. I have made some observations on this point, as follows:

In the early part of August, I selected seven patients in the third stage of consumption. With considerable care I took their temperatures, morning and evening. These observations were made between 8 and 9:30 o'clock a. m.—between 6:30 and 7:30 p. m. The same thermometer was used in all the cases, and was uniformly placed under the tongue.

CASE I.—Hans J., a Norwegian barber. The average of twenty-two consecutive morning observations—Aug. 24 to Sept. 14, both included—was 99.5°; of nineteen consecutive evening observations, 100.24°; making an average diurnal rise of .74°.

The post mortem examination showed a cavity at either apex, surrounded by considerable cheesy consolidation; also a very thick dissemination of miliary tubercles through both lungs, most numerous near the apices, but scattered thickly even to the bases.

CASE II.—Wm. McD., an Irish porter, aged 38 years. The average of twenty-six consecutive morning temperatures—Aug. 24 to Sept. 18, both included—was 99.7°; of twenty-five evening temperatures, 100.6°; giving an average evening rise of .9° F.

The lungs were found to contain each a small cavity at the apex; also considerable cheesy matter in the upper part, and extensive deposits of miliary tubercle throughout.

CASE III.—Jas. S., an English carpenter, 26 years old. The average of thirty-one observations was 100.7° for the morning, and 101.8° for the evening temperature—an average evening rise of 1.1°.

The lungs showed two cavities, cheesy masses, and a considerable sprinkling of miliary tubercles, less than in either of the other cases.

CASE IV.—Lizzie L., an American servant girl, 19 years old.

The average morning temperature from Oct. 4th to 25th, was 100.66° ; the evening, 101.02° ; making an average rise of .36°.

The lungs contained not only cavities, but an abundance of miliary tubercles.

CASE V.—Nils N., a Swedish laborer, 52 years of age. Owing to multiplicity of duties, I took the temperature for only five days. The temperature averaged in the morning 100.06° ; in the evening, 100.87° ; an average evening rise of .81°.

An examination showed an immense cavity in one lung, a small one in the other—both lungs thickly studded with miliary tubercles.

CASE VI.—John B., an Irish tailor. The average of twenty-six morning observations—Aug. 24 to Sept. 18, both included—was 100.13° ; evening, 101.08° ; an average rise of .95°. In this case the ends of science were defeated by the vigilance of friends, who removed the body before an examination was made.

In these cases we observe that the fever was of a continued form—the diurnal variation in five of the six cases being less than one degree—and that the lungs contained miliary tubercles as well as cheesy masses. In my—

CASE VII.—That of John F., an American butcher, the average morning temperature from Aug. 24 to Sept. 18 was 99.16° ; evening, 101.74° ; an average rise of 2.58°. His lungs contained cavities and cheesy masses varying in size from a hazel to a walnut, scattered through the organ, *but no miliary tubercles.* In this case the fever was markedly remittent, and the miliary tubercles were absent.

These are the only cases which I am at present able to present from personal observation. In looking over the records of the hospital, however, I observed that in July last the temperature had been recorded, morning and evening, for seven days, in a case of consumption, by the then House Physician, Dr. Roswell Park. Upon inquiry, I learned that the Doctor had conceived the same idea as myself, but had carried it into execution only in this one case, which he has kindly permitted me to report.

The average morning temperature was 97.9° ; evening, 100.1° ; an average diurnal rise of 2.2°. There was noted at the autopsy an abundance of cheesy masses, but no miliary tubercles—another case of remittent, or rather intermittent, fever without tubercles.

Now, these observations seem to be in accord, so far as they go, with Niemeyer's ideas regarding the significance of temperature in consumption. So far as I could perceive, the thermometer afforded the only means of ascertaining the supervention of tuberculosis upon phthisis. I examined carefully the sputum, as well as the physical signs afforded by the patients referred to. Nowhere could I detect any marked or constant difference between the simple and tuberculous consumptive. And this seems rational; for *all* had consumption—that is, consolidation and disintegration of the lungs, and, as a consequence, all furnished the signs of solidification and excavation. It is true that the respirations vary somewhat; that is, of two patients presenting the same physical signs—that one having a continued fever breathes more frequently than the other with remittent fever. This is well illustrated by two patients at present under observation. Both are American girls, one 18, the other 19 years old. Each has a large cavity at the left apex, with extensive solidification around it, as well as at the other apex. One who has a remittent fever, the daily variation averaging 2½°, breathes twenty-eight times per minute; the other, with a continued fever (daily variation only one-third of one degree), breathes forty-three times per minute—a majority of fifteen respirations per minute in favor of continued fever. I have observed a similar disparity in other cases. But as the frequency of respiration is modified by several influences, it is of but little value as a diagnostic sign.

Now if these ideas be well grounded, their practical value is manifest. Although some patients die of pure inflammatory consumption, such cases are in the minority; the majority present the miliary tubercles in addition to the cheesy masses. Now the tuberculosis usually supervenes, according to the authorities, after consolidation of the lung is evident, and it is the cheesy masses forming this consolidation which constitute the foci of infection whence proceeds the tuberculosis. As Niemeyer tersely states it, "The greatest danger for the majority of consumptives is that they are apt to become tuberculous." In other words, a simple consolidation of the lung, however extensive, nay, even a certain amount of disintegration and excavation, is not necessarily fatal.

Read Nov. 19, 1877.

